# Supplementary material for: Clinical factors associated with rapid treatment of sepsis
Source: PLoS One. 2021 May 6;16(5):e0250923. doi: 10.1371/journal.pone.0250923 (PMC8101717; doi:10.1371/journal.pone.0250923)
Supplement: S1 Appendix — (PDF) [file pone.0250923.s001.pdf]

S1 Appendix – Examples of SHAP Value Representations

Patient A: Age=76, White, Male, Non-Hispanic, Non-Hypotensive,
blood culture ordered, antibiotics administered, initial lactated taken within 3 hour

variable	value	SHAP value	cumulative log(odds)	predictted probability
Heart rate_min	109	0.358	0.358	0.589
DBP_min	79	0.326	0.684	0.665
Bilirubin_inc_initial	8.6	0.313	0.997	0.731
Glasgow Coma Scale_max	0	0.230	1.227	0.773
SBP_min	95	-0.214	1.013	0.734
SpO2_min	100	0.214	1.227	0.773
WBC perc band_initial	10	0.212	1.439	0.808
Heart rate_initial	162	0.195	1.634	0.837
Sodium_initial	132	-0.154	1.480	0.815
INR_initial	2.1	-0.153	1.327	0.790
...remaining 95 variables	132,2.1,...	0.146	1.473	**0.813**

Patient B: Age=87, White, Male, Non-Hispanic, Hypotensive,
blood culture ordered, antibiotics administered, initial lactated taken within 3 hour,
and 2 liter fluid given within 2 hours since initiation

variable	value	SHAP value	cumulative log(odds)	predictted probability
Creatinine_initial	2.02	0.409	0.409	0.601
Urine Output_initial	50	-0.398	0.012	0.503
SBP_min	89	0.394	0.405	0.600
Fibrinoge_initial	357	-0.273	0.133	0.533
MAP_mean	67.7	0.238	0.370	0.592
CO2_max	NA	0.219	0.590	0.643
Glasgow Coma Scale_max	0	0.211	0.801	0.690
Heart rate_initial	95	-0.207	0.594	0.644
Respiratory rate_max	26	0.202	0.796	0.689
Chronic DX_CKD	0	0.192	0.988	0.729
...remaining 96 variables	66.3,0.8,...	-0.023	0.965	**0.724**

For each patient in the example, the "SHAP value" is the change of log odds for the outcome (rapid completion of treatment) for certain variable taking the corresponding "value". The variables are ordered by their SHAP values with "cumulative log(odds)" summing up the overall changes of log odds along the list. The "cumulative log(odds)" is finally transformed to "predicted probability" using sigmoid function, i.e., $1/(1 + exp\ (-x))$.
