# Supplementary material for: Clinical factors associated with rapid treatment of sepsis
Source: PLoS One. 2021 May 6;16(5):e0250923. doi: 10.1371/journal.pone.0250923 (PMC8101717; doi:10.1371/journal.pone.0250923)
Supplement: S2 Appendix — Marginal effects of variables ranked top 11–20 for Model 1 (Panel A) and Model 2 (Panel B) based on SHAP values, i.e. exponential of the SHAP value. (PDF) [file pone.0250923.s002.pdf]

S2 Appendix - Marginal effects of variables ranked top 11 - 20 for Model 1 (Panel A) and Model 2 (Panel B) based on SHAP values, i.e. exponential of the SHAP value.

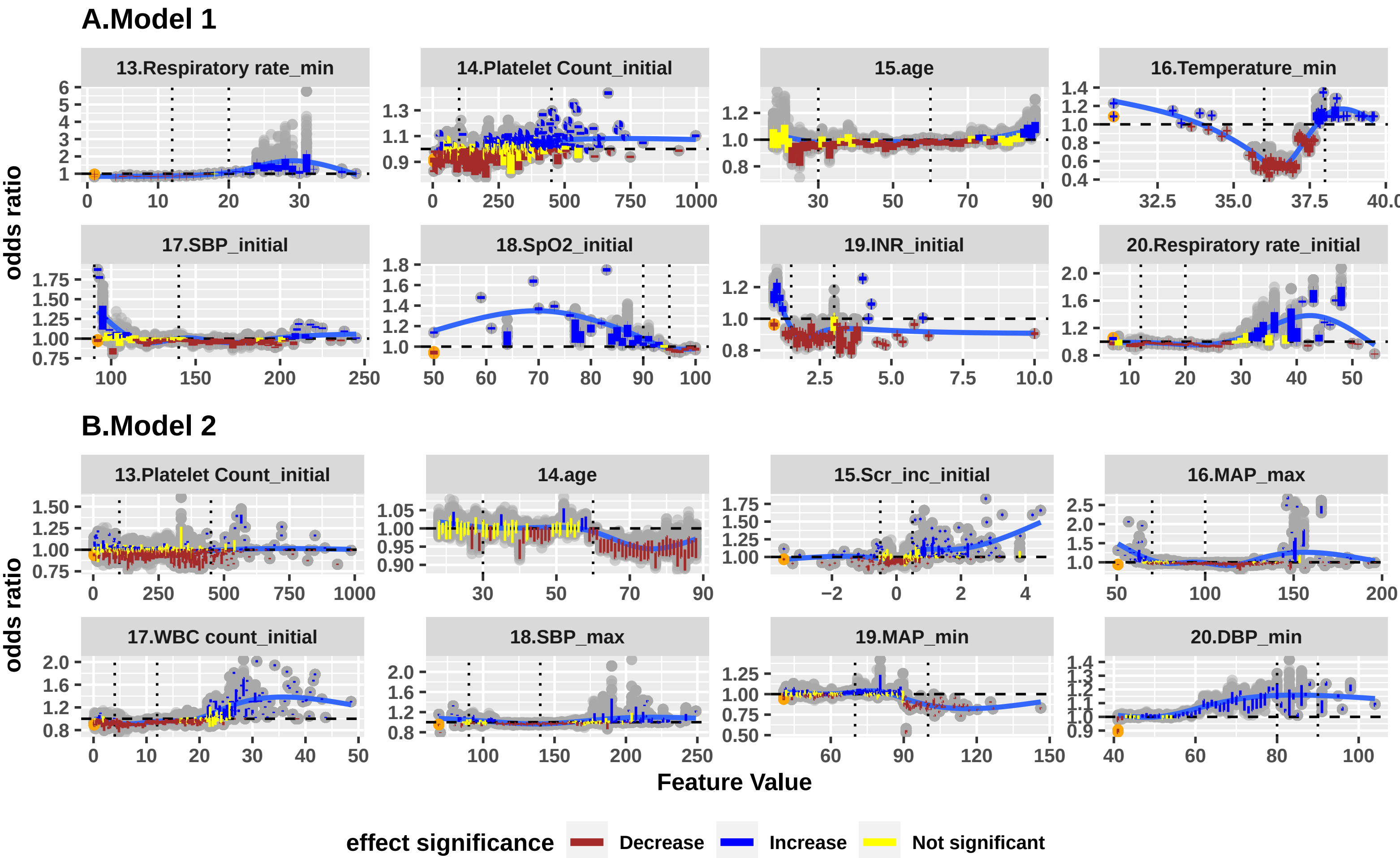

Each dot represents an average change of odds ratio for a variable, taking certain values within a bootstrapped sample. Each colored vertical line depicts a 95% bootstrap confidence interval based on 100 bootstrapped samples. A brown line suggests an odds ratio change significantly higher than 1.0; a blue line suggests an odds ratio change significantly lower than 1.0; a yellow line suggests an odds ratio not significantly different from 1.0. Orange dots represent the odds ratio effect of not having the particular data point recorded for the model. The dashed horizontal line shows an odds ratio of 1.
