# Supplementary material for: Clinical factors associated with rapid treatment of sepsis
Source: PLoS One. 2021 May 6;16(5):e0250923. doi: 10.1371/journal.pone.0250923 (PMC8101717; doi:10.1371/journal.pone.0250923)
Supplement: Attachment — Submitted filename: comments.pdf [file pone.0250923.s003.pdf]

The manuscript applies association rule mining to identify features that are associated with rapid treatment of Spesis patients in Emergency Department (ED). The paper defines an interesting research question, and addresses it using Gradient boosting models on ED visits data. Overall, the manuscript is well-written; however, there are several shortfalls that need to be addressed:

Assumptions and Scope:

Authors mentioned several key assumptions and definitions throughout the paper. As a reader, I would like to see them all at one place, preferably toward the beginning of the paper as bullet-points. Currently, they are defined as needed all over the paper. For example, “Since sepsis, so defined, was considered to be present on admission…” in page 5.

Also, “…components of the 3-hour bundle represent standard elements of excellent sepsis care and have always been present in well-treated patients” in page 5.

Similarly, “We hypothesize that patients with abnormal GCS may slow sepsis treatment, because they first receive …” in page 14.

To set the stage, most of the key limitations in Page 15 (e.g. “definition of suspected infection was based on clinicians’ perceptions of infection”) need to be mentioned earlier, so the readers exactly know what the scopes are.

Method:

Authors used XGboost and SHAP packages in R. Since these are well-known methods, I would have appreciated if you included more details about the modeling process, certain data normalization/standardization procedures for continuous variables, tuning and parameter selection phase, and the reason for not having a testing set (authors used 70% training, and 30% validation sets, split randomly).

I liked the brief juxtaposition of XGBoost and logistic regression on page 13. It is better to have it earlier in the methods section.

Since authors used historical data, it will be interesting to see the distribution of different Diagnosis Related Groups (DRG) or ICD-10 codes among Sepsis population. This can further validate your Sepsis identification method.

Results/Discussion:

Seems like variables related to vital signs play a big role in rapidity of treatment, which is quite expected. Authors also discuss the possibility of using other variables such as machine generated features (e.g. mean respiratory rate over time in page 15), and social factors (e.g. income, education in page 15). Since the latter is the study limitation, I would like to see what the findings of previous research about these features were and how your findings compare to them. In doing so, you may refer to some of the recent similar works, e.g. [1], [2].

Moreover, authors mentioned an important aspect of the subject where operational factors contributing to the ED wait times (e.g. staffing ration, wait time in page 15) can impact physicians' decision to expedite treatments. As far as I know, congested downstream units (e.g. ICU) prolongs the expected ED boarding time. Providers, on the other hand, initiate/expedite the treatment in the ED when they foresee a long wait time. I would like to see a brief discussion on this matter. Please refer to [3] for the case of ICU beds.

Also, you may define the terms "risk factor" and "contributing factor" that were used for features between two different groups.

Minor Comments:

- Page 5. (ref. to SSC document) needs a proper citation
- Page 11. "The marginal effects of the top 20 features for each model are shown in Figure 5 and Figure 6." is repeated a few lines above.
- Page 12, explanations for Figures 5 and 6 are almost identical, so I would merge them together.
- Quality of the provided figures are poor. Maybe this is because of the word-pdf conversion.

References:

[1] P. Palma and J. Rello, "Precision medicine for the treatment of sepsis: recent advances and future prospects," *Expert Rev. Precis. Med. Drug Dev.*, vol. 4, no. 4, pp. 205–213, Jul. 2019.

[2] J. W. Uffen, J. J. Oosterheert, V. A. Schweitzer, K. Thursky, H. A. H. Kaasjager, and M. B. Ekkelenkamp, "Interventions for rapid recognition and treatment of sepsis in the emergency department: a narrative review," *Clin. Microbiol. Infect.*, Feb. 2020.

[3] I. Hasan, E. Bahalkeh, and Y. Yih, "Evaluating intensive care unit admission and discharge policies using a discrete event simulation model," *Simulation*, p. 003754972091474, Apr. 2020.
