# Supplementary material for: Clinical factors associated with rapid treatment of sepsis
Source: PLoS One. 2021 May 6;16(5):e0250923. doi: 10.1371/journal.pone.0250923 (PMC8101717; doi:10.1371/journal.pone.0250923)
Supplement: Attachment — Submitted filename: Response to Comments_final.docx [file pone.0250923.s004.docx]

The manuscript applies association rule mining to identify features that are associated

with rapid treatment of Spesis patients in Emergency Department (ED). The paper

defines an interesting research question, and addresses it using Gradient boosting

models on ED visits data. Overall, the manuscript is well-written; however, there are

several shortfalls that need to be addressed:

**Assumptions and Scope:**

Authors mentioned several key assumptions and definitions throughout the paper. As a

reader, I would like to see them all at one place, preferably toward the beginning of the

paper as bullet-points. Currently, they are defined as needed all over the paper. For

example, “Since sepsis, so defined, was considered to be present on admission…”in

page 5. Also, “…components of the 3-hour bundle represent standard elements of excellent sepsis care and have always been present in well-treated patients” in page 5. Similarly, “We hypothesize that patients with abnormal GCS may slow sepsis treatment, because they first receive …” in page 14. To set the stage, most of the key limitations in Page 15 (e.g. “definition of suspected infection was based on clinicians’ perceptions of infection”) need to be mentioned earlier, so the readers exactly know what the scopes are.

***Response*:** We thank reviewer for the suggestions on reorganizing the paper to achieve better clarity. We have moved the key assumptions and definitions toward the beginning of the paper on page 4 and 5. More specifically,

- We have defined the study cohort using bullet points on page 5.
- the “S*ince sepsis, so defined, was considered to be present on admission*…”on page 5, is now integrated in the exclusion criteria: “*To further exclude patients who were admitted through the ED but developed sepsis later in their hospitalization, i.e. to include only sepsis present on admission, we inferred an hour boundary based on the timing distribution of patients with infection present on admission, which was 13 hours since triage.*” on page 5.
- the “*…components of the 3-hour bundle represent standard elements of excellent sepsis care and have always been present in well-treated patients*” in page 5, is now integrated in: “*The outcome of interest was timely completion of the Surviving Sepsis Guidelines (SSC) 3-hour bundle components (10). We chose the SSC bundles not as an endorsement, but as quantifiable, time-stamped, and recorded actions that are representative of rapid treatment and that are widely known to critical care and emergency practitioners. The specific treatment bundles were not proposed until 2012, but the components of the 3-hour bundle represent standard elements of excellent sepsis care and have always been present in well-treated patients. ..*.” on page 5
- the “*definition of suspected infection was based on clinicians’ perceptions of infection*” on page 15, is now integrated in one of the inclusion criteria “*presence of a suspected infection is based on definition following clinicians’ perceptions of infection, which was defined as a body fluid culture ordered and anti-infective administered within four hours of one another*” described on page 5.

However, the “We hypothesize that patients with abnormal GCS may slow sepsis treatment, because they first receive …” in page 14, was not something we would have known at the design stage. This is one of the findings of the study that we interpreted from a clinical point of view.

**Method:**

Authors used XGboost and SHAP packages in R. Since these are well-known methods,

I would have appreciated if you included more details about the modeling process,

certain data normalization/standardization procedures for continuous variables, tuning

and parameter selection phase, and the reason for not having a testing set (authors

used 70% training, and 30% validation sets, split randomly). I liked the brief juxtaposition of XGBoost and logistic regression on page 13. It is better

to have it earlier in the methods section. Since authors used historical data, it will be interesting to see the distribution of different Diagnosis Related Groups (DRG) or ICD-10 codes among Sepsis population. This can further validate your Sepsis identification method.

***Response*:** We have added more technical details regarding modeling process, data preprocessing and tuning in the Method section (page 6). Note that to retain better interpretation of modeling results as well as attribute to the robustness of tree-based models such as GBM, we didn’t perform additional normalization/standardization procedures for continuous variables, except for missing value handling. We’ve also mentioned the comparison with logistic regression ahead of time.

With regard to ICD coding, this could, indeed be interesting, but probably does not validate the technique. In fact we used a method of sepsis identification very similar to that of Rhee, et al, (JAMA, 2017). Additionally, we demonstrated in a related dataset from our institution that ICD codes are quite insensitive for identifying patients with infection-induced organ dysfunction (Deis, et al. CHEST, 2017) The work would be interesting, but we don’t believe that it would enhance our findings.

**Results/Discussion:**

Seems like variables related to vital signs play a big role in rapidity of treatment, which

is quite expected. Authors also discuss the possibility of using other variables such as

machine generated features (e.g. mean respiratory rate over time in page 15), and

social factors (e.g. income, education in page 15). Since the latter is the study limitation,

I would like to see what the findings of previous research about these features were and

how your findings compare to them. In doing so, you may refer to some of the recent

similar works, e.g. [1], [2].

Moreover, authors mentioned an important aspect of the subject where operational

factors contributing to the ED wait times (e.g. staffing ratio, wait time in page 15) can

impact physicians’ decision to expedite treatments. As far as I know, congested

downstream units (e.g. ICU) prolongs the expected ED boarding time. Providers, on the other hand, initiate/expedite the treatment in the ED when they foresee a long wait time.

I would like to see a brief discussion on this matter. Please refer to [3] for the case of

ICU beds. Also, you may define the terms “risk factor” and “contributing factor” that were used for features between two different groups.

***Response*:** We thank reviewers for this comment. [1] suggested the importance of personalized medicine advancement in helping better diagnose and treat sepsis patients, which is exactly something we are trying to achieve using a robust machine learning model. However, [1] emphasized on discovering genomic biomarkers, while we focused more on phenotypic features that are commonly recorded in EMR data. The majority of our findings involve vital signs. Although that the information might have been anecdotally predicted, we believe this to be the first study quantifying the associations of vital signs data with rapidity of treatment on a continuous scale. In [2], it pointed out that “most studies reported on changes in compliance with bundle elements and/or mortality rates”, however, few of those studies took a step further to understand risk factors that affect the compliance or non-compliance, which is something we are trying to uncover here. To emphasize on this point, we have added the following paragraph in the discussion section: “As discussed in [2], awareness and compliance with protocols can be difficult to maintain in the absence of an effective clinical pathway, which requires careful planning and dedicated resources. With a better understanding of alerting features associated with rapid treatment would provide useful and evidence-based insights to a better design for creating such clinical pathway and continuous education. ”.

It is an interesting proposition that treatment is initiated or expedited in the ED when ED personnel foresee a long wait time. This would be true under ideal circumstances; however, in the real time workflow of the emergency department and the hospital floors and ICUs, it is rare that one can actually foresee the circumstance. Beds can and do open at any time, and patients are accepted by the admitting team, whether on the hospital ward or in the ICU, as soon as they can be. It is rare, certainly in our hospital, that the ED physicians can actually anticipate that there will be a long delay in transfer to a floor bed or ICU bed. The variable and unpredictable nature of bed availability leads to variable behavior on the part of ED physicians. The simulation you reference [3] necessarily assumes that there is a rational, predictable behavior that simply does not exist under live circumstances. In fact, a full ICU can and does lead to later implementation of sepsis bundles, once time has passed and the ED physician realizes that the patient will not be moving soon from the ED. Delays in this circumstance can be significant.

**Minor Comments:**

- Page 5. (ref. to SSC document) needs a proper citation

- Page 11. “The marginal effects of the top 20 features for each model are shown in Figure 5 and Figure 6. is repeated a few lines above.

- Page 12, explanations for Figures 5 and 6 are almost identical, so I would merge

them together.

- Quality of the provided figures are poor. Maybe this is because of the word-pdf

conversion.

***Response*:** We thank reviewer for the careful review! We have addressed all the minor comments and included figures of better resolution. In terms of the pictures, we have tried to save raw format as .tiff files which should guarantee better resolutions. Please also note that we have merged Figure 5 and 6 as new Figure 5, and make Figure 7 as new Figure 6.

**References:**

[1] P. Palma and J. Rello, “Precision medicine for the treatment of sepsis: recent

advances and future prospects,” *Expert Rev. Precis. Med. Drug Dev.*, vol. 4, no.

4, pp. 205–213, Jul. 2019.

[2] J. W. Uffen, J. J. Oosterheert, V. A. Schweitzer, K. Thursky, H. A. H. Kaasjager,

and M. B. Ekkelenkamp, “Interventions for rapid recognition and treatment of

sepsis in the emergency department: a narrative review,” *Clin. Microbiol. Infect.*,

Feb. 2020.

[3] I. Hasan, E. Bahalkeh, and Y. Yih, “Evaluating intensive care unit admission and

discharge policies using a discrete event simulation model,” *Simulation*, p.

003754972091474, Apr. 2020.
