# Supplementary material for: Clinical factors associated with rapid treatment of sepsis
Source: PLoS One. 2021 May 6;16(5):e0250923. doi: 10.1371/journal.pone.0250923 (PMC8101717; doi:10.1371/journal.pone.0250923)
Supplement: Attachment — Submitted filename: Response to Reviewers_rev2.docx [file pone.0250923.s005.docx]

Review Comments to the Author

Please use the space provided to explain your answers to the questions above. You may also include additional comments for the author, including concerns about dual publication, research ethics, or publication ethics. (Please upload your review as an attachment if it exceeds 20,000 characters)

Reviewer #1: * The figure's resolution is much better now.
* Please don't use acronyms before defining them, such as ED in the abstract. Don't assume all the readers familiar with these acronyms, even if there are popular.
* Add similar research with SHAP and Sepsis or other complications. Also, studies for predicting Sepsis or other complications. Write more about it in the introduction. Compare and discuss the results with other studies, write on the differences.

Here some suggestions for quite similar studies:

Murugesan, I., Murugesan, K., Balasubramanian, L., & Arumugam, M. (2019, September). Interpretation of Artificial Intelligence Algorithms in the Prediction of Sepsis. In 2019 Computing in Cardiology (CinC) (pp. Page-1). IEEE.

Lauritsen, S. M., Kristensen, M., Olsen, M. V., Larsen, M. S., Lauritsen, K. M., Jørgensen, M. J., ... & Thiesson, B. (2020). Explainable artificial intelligence model to predict acute critical illness from electronic health records. Nature communications, 11(1), 1-11.

Yang, M., Liu, C., Wang, X., Li, Y., Gao, H., Liu, X., & Li, J. (2020). An Explainable Artificial Intelligence Predictor for Early Detection of Sepsis. Critical Care Medicine, 48(11), e1091-e1096.

Komorowski, M., Celi, L. A., Badawi, O., Gordon, A. C., & Faisal, A. A. (2019). Understanding the Artificial Intelligence Clinician and optimal treatment strategies for Sepsis in intensive care. arXiv preprint arXiv:1903.02345.

And similar papers for prediction of complications:

Reyna, M. A., Josef, C., Seyedi, S., Jeter, R., Shashikumar, S. P., Westover, M. B., ... & Clifford, G. D. (2019, September). Early prediction of Sepsis from clinical data: the PhysioNet/Computing in Cardiology Challenge 2019. In 2019 Computing in Cardiology (CinC) (pp. Page-1). IEEE.

Fleuren, L. M., Klausch, T. L., Zwager, C. L., Schoonmade, L. J., Guo, T., Roggeveen, L. F., ... & Elbers, P. W. (2020). Machine learning for the prediction of Sepsis: a systematic review and meta-analysis of diagnostic test accuracy. Intensive care medicine, 1-18.

Itzhak, N., Nagori, A., Lior, E., Schvetz, M., Lodha, R., Sethi, T., & Moskovitch, R. (2020, August). Acute Hypertensive Episodes Prediction. In International Conference on Artificial Intelligence in Medicine (pp. 392-402). Springer, Cham.

Mao, Q., Jay, M., Hoffman, J. L., Calvert, J., Barton, C., Shimabukuro, D., ... & Das, R. (2018). Multicentre validation of a sepsis prediction algorithm using only vital sign data in the emergency department, general ward and ICU. BMJ open, 8(1).

Cherifa, M., Blet, A., Chambaz, A., Gayat, E., Resche-Rigon, M., & Pirracchio, R. (2020). Prediction of an acute hypotensive episode during an ICU hospitalization with a super learner machine-learning algorithm. Anesthesia & Analgesia, 130(5), 1157-1166.

Response to Reviewer 1: We appreciate reviewer’s comments for the minor revision.

- We have identified all acronyms and added their full spellings when first introduced in the text. In addition, we have added a new “Acronym” section at the beginning of the text.
- We have also enriched the “Introduction” section with all the recommended articles added. On second page, we added “…Numerous efforts have been made to improve prognostic accuracy and efficiency for sepsis and its complications via machine learning techniques. For example, Yang et al.(8), Komorowski et al. (9) and Reyna et al. (10) developed artificial intelligence models to predict sepsis in intensive care; while Mao et al. (11) and Lauritsen et al. (12) extended the prediction application to ED and general ward. Itzhak et al. (13) and Cherifa et al. (14) developed models to predict acute hypertensive or hypotensive episodes among ICU admissions. However, few, if any studies have been designed to understand specific clinical features that patients exhibit at the time that physicians initiate rapid treatment of sepsis. Without assuming causality, one could evaluate from a situational awareness perspective which clinical features are most closely associated with rapid, thorough treatment. We believed that data from such a study could provide novel information that could be used to prompt rapid sepsis treatment for appropriate patients, regardless of the extant diagnostic criteria.”

Reviewer #2: (No Response)

Reviewer #3: In the revised manuscript titled "Clinical Factors Associated with Rapid Treatment of Sepsis", the authors improved dramatically the writing. It is now much clearer, and the authors addressed all previous comment and suggestion raised by the reviewer.
However I still have a few comments which can improve the manuscript farther.
- The author listed 3 criteria "Patients were included by satisfying the following criteria:" Are all three criteria required for every sample to be included?
- "EMS personnel" abbreviation is used w/o definition.
- Table 2:
1. There is no need for replicating column titles for every subsection.
2. I would recommend to stratify columns also by rapid treatment vs not.
- Figure 6: It is not clear whether this is for group 2 only, or cross sectional.

Response to Reviewer 3: We appreciate reviewer’s comments for the minor revision.

- Yes. All three criteria are required for the patient encounter to be eligible for the study, and we have stated so very specifically in this revision.
- EMS stands for Emergency Medical Service, besides EMS, we have identified all acronyms and added their full spellings when first introduced in the text. In addition, we have added a new “Acronym” section at the beginning of the text.
- For Table 2, we believe we understand what the reviewer is intending, and we considered altering the table. However, on reflection, we believe that we should leave the table as is. The main thrust of the paper is to identify the demographic and other characteristics that identify, i.e. separate, the patients who receive rapid treatment, compared with those who do not. We do so with much more sophisticated techniques than one would employ with a perusal of summary statistics in a table. We opted to provide the demographics of the entire study set and to look at the subsets of those who required vasopressors and those who did not, and we think this better serves the purpose of informing the reader of who our study population is. This is underscored by the fact that demographic information was not among the features with the greatest effect in the model. The physiological data that most significantly separate rapidly treated patients from others are displayed in Figure 2, while demographics do not rise to that level of influence. We did remove the redundant column titles, as suggested.
- Figure 6 is on the entire or cross-sectional data set, i.e. for both group 1 and group 2.
